# Long-Term Clinical and Endoscopic Outcomes of Crohn’s Disease Following Liver Transplantation: A Multicenter Cohort Study

**DOI:** 10.3390/biomedicines13092200

**Published:** 2025-09-08

**Authors:** Ebru Ar, Irini Solomonidou, Henrike Lenzen, Miriam Wiestler, Claudia Veltkamp, Katharina Willuweit, Jassin Rashidi-Alavijeh, Hartmut H. Schmidt, Richard Vollenberg, Phil-Robin Tepasse, Jonel Trebicka, Stefanie Tischendorf, Carsten Elfers, Karim Hamesch, Arne Bokemeyer

**Affiliations:** 1 Department of Gastroenterology, Hepatology, and Transplant Medicine, University Hospital Essen, Faculty of Medicine, University of Duisburg-Essen, 45147 Essen, Germany; ebru.ar@uk-essen.de (E.A.); claudia.veltkamp@uk-essen.de (C.V.); katharina.willuweit@uk-essen.de (K.W.); jassin.rashidi@uk-essen.de (J.R.-A.); hartmut.schmidt@uk-essen.de (H.H.S.); 2 Department of Medicine B (Gastroenterology, Hepatology, Endocrinology, Clinical Infectiology), University Hospital Muenster, 48149 Muenster, Germany; solomoni@web.de (I.S.); richard.vollenberg@ukmuenster.de (R.V.); phil-robin.tepasse@ukmuenster.de (P.-R.T.); jonel.trebicka@ukmuenster.de (J.T.); 3 Department of Gastroenterology, Hepatology, Infectious Diseases, and Endocrinology, Hannover Medical School, 30625 Hannover, Germany; h.lenzen@skbs.de (H.L.); wiestler.miriam@mh-hannover.de (M.W.); 4 Medical Clinic III, Gastroenterology, Metabolic Diseases, and Intensive Care, University Hospital RWTH Aachen, 52074 Aachen, Germany; stischendorf@ukaachen.de (S.T.); celfers@ukaachen.de (C.E.); khamesch@ukaachen.de (K.H.); 5 Department for Gastroenterology, Diabetology, and Palliative Care, Bonifatius Hospital Lingen, 49808 Lingen, Germany

**Keywords:** liver transplantation, Crohn’s disease, advanced therapies, biologics, immunosuppression

## Abstract

Patients with Crohn’s disease (CD) may require liver transplantation (LT) due to advanced liver diseases, including primary sclerosing cholangitis (PSC), autoimmune hepatitis, or other etiologies. However, data on CD activity and the use of advanced therapies following LT are limited. This study aimed to assess CD activity before and after LT and to evaluate the use of advanced therapies in this setting. **Methods:** In this multicenter retrospective cohort study, we analyzed 40 patients with CD who underwent LT between 2000 and 2022 at four university hospitals in Germany. Clinical and endoscopic disease activity, as well as the use of advanced therapies, were evaluated before and after transplantation. **Results:** Before LT, 89.7% of patients were in clinical remission, which remained stable after LT (85.7%; *p* = 0.650). Nevertheless, 22.6% of these patients demonstrated moderate to severe mucosal inflammation on endoscopy during long-term follow-up. The use of advanced therapies remained low after transplantation (pre-LT: 11.7%, post-LT: 7.5%; *p* = 0.532) even among those with endoscopic disease activity. **Conclusions:** Although clinical remission of CD is usually maintained following LT, endoscopic evidence of persistent mucosal inflammation is common and may be underrecognized. Despite this, advanced therapies are not frequently used in the post-transplant setting. These findings suggest that individualized treatment strategies are needed to address subclinical disease activity while balancing therapeutic effectiveness with transplant-specific risks.

## 1. Introduction

Crohn’s disease (CD) is a chronic, relapsing inflammatory disorder of the gastrointestinal tract, most commonly presenting with abdominal pain, diarrhea, and other digestive symptoms. Inflammatory bowel diseases (IBDs), including CD, are increasingly recognized as systemic conditions that may affect organ systems beyond the gut [1,2]. Among these extraintestinal manifestations, liver involvement is among the most clinically relevant, with a range of hepatic pathologies described in affected individuals [3,4].

Among these, primary sclerosing cholangitis (PSC) is a progressive cholestatic liver disease that occurs in approximately 1–3% of patients with CD and constitutes a small but clinically important subset [5,6,7]. PSC is characterized by diffuse inflammation and fibrotic narrowing of the biliary tree, progressing to biliary cirrhosis in 40% of patients within 15 years [8]. In advanced stages, PSC and other hepatic comorbidities—such as autoimmune hepatitis, hepatic amyloidosis, viral hepatitis, and inherited metabolic disorders—may result in irreversible liver damage requiring transplantation (LT) [9].

Although LT is the definitive treatment for end-stage liver disease, the complex relationship between immunosuppressive regimens, pre-existing disease activity, and the use of advanced therapies (i.e., monoclonal antibodies and small molecules) in patients with CD is not well understood. Immunosuppression is essential to prevent graft rejection but may have unpredictable effects on intestinal inflammation. Previous data, mostly from small, single-center studies, suggest considerable heterogeneity in post-LT disease trajectories: while some patients achieve sustained remission, others experience persistent or worsening inflammation [10,11]. Most earlier studies, however, have focused exclusively on ulcerative colitis (UC) or IBD more broadly; none have examined CD in isolation [10,11].

Unlike UC, for which colectomy can offer a curative option, CD has no surgical equivalent. Its management, therefore, relies primarily on advanced therapies that support sustained inflammatory control and help reduce long-term complications [12,13].

Over the past two decades, medical treatment options for CD have expanded considerably with the introduction of biologics and small molecules that target specific inflammatory pathways [14].

Despite the relevance of these agents to disease control, their safety and utility in LT recipients remain poorly characterized [15]. Most available studies predate the widespread adoption of advanced therapies and provide limited guidance for contemporary clinical decision-making.

To address this gap, we conducted a multicenter, retrospective cohort study of patients with CD who underwent LT. The aim was to characterize clinical and endoscopic disease activity before and after LT and to evaluate the real-world use and safety of advanced therapies in this setting. The findings are intended to point towards future directions in the management of CD after LT.

## 2. Materials and Methods

### 2.1. Study Design and Setting

This retrospective, observational cohort study was conducted across four university-affiliated tertiary care centers in Germany, each with dedicated hepatology and IBD services. The study period extended from January 2000 to December 2022. The final cohort comprised 40 patients with CD who underwent LT during this period.

The study was approved by the Ethics Committee of the Medical Faculty of Essen University Hospital (approval no. 23-11173-BO) on 21 March 2023. Informed consent was not required due to the retrospective nature of the study, in accordance with local ethical guidelines.

### 2.2. Data Collection

Adult patients (≥18 years) with a confirmed diagnosis of CD who underwent LT were identified using electronic medical records. Data extraction was performed using standardized procedures. For each patient, the following information was recorded: demographic and biochemical parameters (age, sex, C-reactive protein [CRP], and fecal calprotectin [fCal] levels); clinical history (date of initial CD diagnosis; pre-transplant data, including previous CD-related surgeries or interventions); CD-specific therapies, including the use of advanced therapies (biologics and small molecules); and bowel-related endoscopic findings. Hepatological data included liver-related diagnoses and treatment regimens. CD activity and the occurrence of disease flares were recorded for the periods before, during, and after LT. Patients with ulcerative colitis, indeterminate colitis, or insufficient documentation of IBD-related variables were excluded.

Clinical disease activity was assessed using patient-reported outcome measures, consistent with previously published criteria [16,17,18]. Clinical remission was defined as a mean daily stool frequency (SF) of ≤3 over one week and an abdominal pain score of ≤1, according to the PRO2 definition [16,17,18]. Endoscopic disease activity was assessed using a semi-quantitative score ranging from 0 (normal) to 3 (severe) to grade mucosal inflammation [11].

Each patient was evaluated individually to monitor disease progression and assess treatment outcomes across predefined time intervals. Clinical data were systematically recorded using a customized electronic spreadsheet and categorized into three distinct phases: pre-transplant, post-transplant, and long-term follow-up. These intervals were operationally defined as follows: the pre-transplant period comprised the 3 to 6 months before LT; the post-transplant period, 3 to 18 months after LT; and the long-term follow-up phase referred to assessments conducted beyond 18 months after LT. For each phase, the most recent available clinical evaluation was selected for analysis. In addition to demographic and baseline clinical parameters, disease activity was quantified using established IBD activity scores, calculated separately for each interval.

Severe infectious complications were systematically recorded and defined as infections necessitating inpatient hospitalization, surgical intervention, or those associated with mortality.

### 2.3. Statistical Analysis

Statistical analyses were conducted using IBM SPSS Statistics, version 22 (IBM Corp., Armonk, NY, USA). Categorical variables were analyzed using contingency tables, with supplementary calculations performed via the StatPages online platform. Descriptive statistics were used to summarize baseline characteristics, clinical parameters, and outcome measures. Comparisons between pre- and post-transplant data were made using chi-squared tests or Fisher’s exact test for categorical variables and *t*-tests for continuous variables, as appropriate. To reduce the risk of bias, data collection procedures were standardized across all participating centers. Predefined eligibility criteria were applied to minimize selection bias, and all data were abstracted by trained reviewers following a uniform protocol to ensure consistency and data integrity.

## 3. Results

### 3.1. Baseline Characteristics

A total of 40 patients with CD who met the inclusion criteria and underwent LT between 2000 and 2022 were included in the final analysis (Table 1). The mean age was 27.3 years (standard deviation [SD] ± 12.4) at initial diagnosis and 41.2 years (SD ± 14.5) at the time of LT. The cohort consisted of 25 male (62.5%) and 15 female (37.5%) patients. At diagnosis, disease extent was classified as ileocolonic (L3) involvement in 46.2% of patients, with an additional 2.6% exhibiting concomitant upper gastrointestinal tract involvement (L3 + L4). Isolated colonic (L2) and ileal (L1) involvement were each observed in 25.6% of cases. Most patients (92.5%) received a graft from a deceased donor, and 7.5% underwent transplantation from a living donor. The most frequent indication for LT was PSC (52.5% of cases), followed by hepatic overlap syndrome (17.5%), cryptogenic cirrhosis (10%), autoimmune hepatitis (7.5%), viral hepatitis (5%), metabolic dysfunction-associated steatohepatitis (5%), and other causes (2.5%) (Table 1).

### 3.2. Crohn’s Disease Activity During Pre-Transplantation Period

The mean daily SF over one week was 1.36 (±1.87). Abdominal pain was reported by 31% of patients with mild or moderate pain intensity, while 69% reported no pain. Clinical remission was achieved in 89.7% of patients prior to LT. Before transplantation, 40% of patients received corticosteroids and aminosalicylates (35.3%). Advanced therapies were used in only a minority of cases (11.7%) (Table 2 and Table 3).

### 3.3. Crohn’s Disease Activity During Post-Transplantation Period

**Figure 1:**
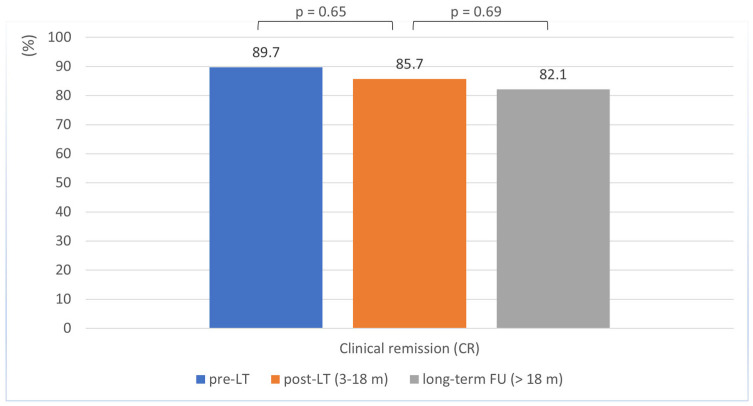
Clinical remission in Crohn’s disease patients before, 3–18 months after, and during long-term follow-up after liver transplantation.

Clinical remission rates remained stable at 85.7% during the 3–18 months following LT, with no significant difference from the pre-LT rate of 89.7% (*p* = 0.650) (Table 2, Figure 1). No age-related differences in clinical remission rates were observed (<40 years vs. ≥40 years; *p* = 0.846; Supplemental Table S1).

### 3.4. Crohn’s Disease Activity During Long-Term Follow-Up

At long-term follow-up (>18 months post-transplantation; mean duration: 93 months [SD ± 71.7]), most patients remained in clinical remission, although the remission rate showed a slight decline from 85.7% to 82.1%, which was not statistically significant (*p* = 0.690) (Table 2; Figure 1). Here, too, there was no evidence of age-related differences in clinical remission rates (<40 years vs. ≥40 years; *p* = 0.466; Supplemental Table S1).

Among patients with available endoscopic evaluations, 45.2% had normal mucosal findings, while mild endoscopic disease activity was observed in 32.3%. Moderate to severe endoscopic disease activity was present in 22.6% of cases (Table 2 and Table 4).

### 3.5. Use and Safety of CD-Specific Advanced Therapies and Occurrence of Infectious Complications After LT

**Figure 2:**
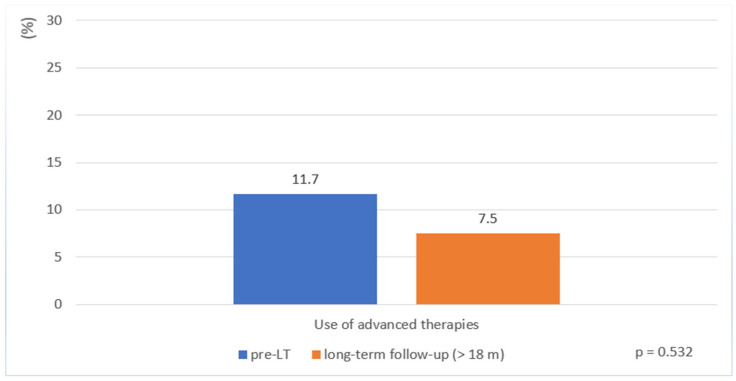
Use of advanced therapies in patients with Crohn’s disease before liver transplantation and during long-term follow-up (>18 months post-transplantation).

Before LT, 11.7% of patients received advanced therapies. Despite persistent moderate to severe mucosal inflammation in a subset of patients, the proportion of those who received an advanced therapy during long-term follow-up remained low (7.5%, *p* = 0.532) (Table 3, Figure 2). These consisted of anti-TNF agents (5%) or vedolizumab (2.5%) (Table 3).

Severe infections requiring hospitalization, surgical intervention, or resulting in death were recorded throughout the follow-up period. All patients receiving an advanced therapy alongside LT-related immunosuppressive treatment (three out of three patients) experienced an infectious complication: two cases of acute cholangitis and a single case of pneumonia. Among patients not receiving IBD-specific advanced therapy, 38.9% (14/36) developed a severe infection, most frequently acute cholangitis (64%) and cytomegalovirus infection (21%). The use of advanced therapies in combination with immunosuppression was not associated with a statistically significant increase in infectious complications (*p* = 0.0744). However, given the low patient numbers, the significance of this aspect needs to be discussed further.

## 4. Discussion

This multicenter retrospective cohort study provides one of the most detailed assessments to date of CD activity in patients undergoing LT, most commonly due to PSC. To our knowledge, this is the largest and most recent analysis of clinical trajectories and the use of advanced IBD therapies in CD patients following LT.

### 4.1. Disease Activity in Patients with Crohn’s Disease Before and After Liver Transplantation

A key finding of this study is the consistency in disease activity observed in CD patients before and after LT. Remission rates were high before LT (89.7%) and similar after transplantation (85.7%), with no statistically significant difference (*p* = 0.650). In long-term follow-up, a slight decline was observed, but this was not significant (82.1%; *p* = 0.69).

These findings are consistent with earlier studies, which, although limited by smaller sample sizes and heterogeneous populations, reported that IBD often remains stable after LT. For example, a review [11] summarizing findings from 15 case series (*n* = 609) reported that approximately one-third of IBD patients experienced clinical improvement after LT, one-third maintained stable disease activity, and the remaining third experienced clinical deterioration. A more recent synthesis including 25 case series and interventional studies found that 81% of patients with IBD had either improved or unchanged clinical status after LT [19]. Additional recent studies have further supported these findings, indicating that IBD, including CD, can in many cases be well controlled following LT [20,21].

Importantly, our study focuses exclusively on CD, enabling a more precise evaluation of disease behavior in this distinct subgroup. The long-term maintenance of remission observed here reinforces previous evidence while expanding the dataset considerably with a more extensive and recent multicenter cohort. It also suggests that LT itself does not inherently worsen CD activity.

Of interest, recent experimental work with mouse models of colitis and sclerosing cholangitis suggests that PSC may attenuate intestinal inflammation in IBD [22]. Following this hypothesis, the loss of such a modulatory effect after LT may contribute to the slight decline in clinical remission rates observed during long-term follow-up. While the observation is speculative, the putative mechanism merits further investigation.

### 4.2. Mucosal Inflammation and the Role of Endoscopy

In line with evolving treatment goals in IBD, mucosal healing is now regarded as an important therapeutic target [23,24,25]. Historically, studies of IBD after LT have focused mostly on clinical symptoms rather than endoscopic disease activity [11]. Our study is among the first in this setting to systematically assess endoscopic outcomes in addition to clinical outcomes with PRO2 (described in Section 4.1).

We defined remission based on endoscopic findings, which are the current gold standard of disease assessment. Consistent with STRIDE II, endoscopic healing is considered a long-term therapeutic goal, whereas biochemical markers such as fCal and CRP are regarded as intermediate targets, and systematic histology has not yet been formally established as a treatment endpoint [24].

During long-term follow-up (>18 months post-LT), approximately 80% of patients remained in clinical remission, yet 55% showed signs of mucosal inflammation on endoscopy. These findings point to possible subclinical disease activity in this population, emphasizing the importance of routine endoscopic surveillance regardless of symptomatic status. This approach is consistent with emerging guidelines recommending the detection and treatment of asymptomatic mucosal inflammation to prevent complications such as flares and colorectal neoplasia [26,27,28].

### 4.3. Use and Safety of Advanced Therapies After Liver Transplantation

Another key finding of our study is the low use of CD-specific advanced therapies before and after LT. Despite evidence of a high rate of persistent mucosal inflammation (55%), only three patients (7.5%) received an advanced therapy such as an anti-TNF or more gut-specific agent like vedolizumab during long-term follow-up. This discrepancy likely reflects ongoing uncertainty among physicians regarding the safety of biologic and small-molecule therapies in transplant recipients. Current data on the use of advanced therapies in IBD patients following LT is sparse [29,30,31]. However, newer agents, such as vedolizumab, ustekinumab, and IL-23-antagonists, may offer advantages due to their gut-selective or more targeted mechanisms of action [32,33,34]. Their use may help achieve mucosal healing in this complex group of patients, although safety concerns persist [24,26,35] due to the additive effects of immunosuppression to prevent transplant rejection. It is also conceivable that the immune-mediated interaction between PSC and CD may contribute to lower intestinal disease activity after LT, reducing the need for treatment escalation. Preclinical studies suggest that inflammatory activity in one organ system (e.g., the liver) may attenuate disease in the other (e.g., the intestine), although the underlying mechanisms remain to be clarified [22]. Regardless of the underlying explanation, the low use of CD-specific advanced therapies before and after LT in our study emphasizes the importance of further research to assess the long-term safety and effectiveness of these newer agents in different patient populations.

All three patients who received advanced therapies during long-term follow-up experienced an infectious complication: two developed cholangitis—a frequent complication in LT recipients that might be related to the type of surgical biliary anastomosis—and one developed pneumonia. Whether these events were directly attributable to advanced therapy remains unclear. Among patients not receiving advanced therapy, 38.9% developed severe infections, most frequently acute cholangitis and cytomegalovirus infection. No statistically significant difference in infection risk was observed between these two groups (*p* = 0.0744), suggesting that advanced therapy may be considered in selected patients with appropriate clinical oversight. However, given the small sample size and retrospective design of our study, these results should be interpreted with care. Our findings are nevertheless consistent with recent reports suggesting that advanced therapies can be used safely in transplant recipients, although further research is needed [29,36,37].

It is also important to consider that the type of baseline immunosuppression used to prevent graft rejection may influence IBD activity. Some studies have suggested that dual immunosuppression with tacrolimus and mycophenolate mofetil may be associated with IBD flares [38], whereas regimens based on cyclosporin and azathioprine may have a more favorable effect [14]. Selecting compatible immunosuppressive and advanced IBD treatment regimens may therefore be critical to optimizing outcomes.

Although combination strategies can increase the likelihood of achieving treatment goals, they are also associated with a higher risk of adverse events. In this setting, individualized treatment should aim to balance effectiveness and safety by prioritizing agents with highly selective mechanisms of action, tailored to each patient’s clinical status, prior treatment history, and cumulative immunosuppressive burden. Such an approach may allow for adequate control of intestinal inflammation while minimizing the overall risk of treatment-related complications [37,39].

### 4.4. Limitations and Strengths

Like all retrospective studies, the present work is subject to methodological limitations, including the risk of selection and reporting bias. However, the relatively large cohort and multicenter design improve the robustness and generalizability of the findings. To our knowledge, this is the largest study to date focusing exclusively on CD patients after LT. In contrast to earlier reports [11,20], which were limited by small sample sizes and a narrow focus on clinical symptoms, our study provides a more comprehensive evaluation of post-LT disease behavior by analyzing clinical, endoscopic, and treatment-related data over an extended follow-up period. It reports new evidence on long-term clinical remission and persistent mucosal inflammation, both of which are areas that have been insufficiently addressed in previous research. As noted above, the small sample size of our study inevitably limits the strength of conclusions that can be drawn regarding the safety profile of the investigated therapies.

### 4.5. Future Directions in the Management of Crohn’s Disease After Liver Transplantation

Our findings emphasize the importance of long-term gastroenterological follow-up in CD patients after LT, including regular endoscopic surveillance regardless of symptomatic status. Given the frequency of subclinical mucosal inflammation, a strong focus on early detection and timely initiation of advanced therapies may be warranted, even in asymptomatic patients. Further studies are needed to evaluate the safety and effectiveness of agents such as ustekinumab, IL-23 inhibitors, and JAK inhibitors in the post-LT population [37,40]. Effective management will require close coordination between gastroenterologists, hepatologists, and transplant specialists to balance disease control with infection and other immunosuppression-related risks. Prospective studies are needed to establish evidence-based treatment guidelines for this complex population [41].

## 5. Conclusions

This multicenter analysis suggests that while clinical remission in CD is frequently maintained during long-term follow-up after LT, endoscopic evidence of disease activity persists in approximately half of patients. Despite the established efficacy of biologics and small molecules in achieving therapeutic goals such as mucosal healing, these agents are used in only a small subset of post-transplant patients with CD. This apparent underutilization, despite ongoing mucosal inflammation, indicates a need for individualized treatment strategies prioritizing agents with highly selective mechanisms of action, well-coordinated multidisciplinary care, and prospective studies to inform evidence-based management in this complex patient population.

## Figures and Tables

**Table 1:** Baseline characteristics of Crohn’s disease patients undergoing liver transplantation.

Variable	Patients (*n* = 40)
Sex	
Male, *n* (%)	25 (62.5)
Female, *n* (%)	15 (37.5)
Inflammatory bowel disease	
Available data, *n* (%)	39 (97.5)
Age at initial CD diagnosis, mean (±SD) years	27.3 (±12.43)
Disease location at diagnosis (Montreal classification), *n* (%)	
L3 (ileocolonic)	18 (46.2)
L1 (ileal)	10 (25.6)
L2 (colonic)	10 (25.6)
L3 + L4 (ileocolonic + upper GI)	1 (2.6)
Liver transplantation	
Age at time of LT, mean (±SD) years	41.23 (±14.5) [*n* = 30]
Type of transplantation, *n* (%)	
Deceased donor	37 (92.5)
Living donor	3 (7.5)
Indications for transplantation, *n* (%)	
Primary sclerosing cholangitis	21 (52.5)
Hepatic overlap syndrome	7 (17.5)
Cryptogenic liver cirrhosis	4 (10)
Autoimmune hepatitis	3 (7.5)
Viral hepatitis	2 (5)
Metabolic dysfunction-associated steatohepatitis	2 (5)
Other	1 (2.5)

**Table 2:** Clinical Crohn’s disease activity before, 3–18 months after, and during long-term follow-up after liver transplantation (LT).

	Before LT (3–6 m Pre-LT)		After LT (3–18 m Post-LT)		Long-Term Follow-Up (>18 m Post-LT)	
Continuous Variables	Mean Value ± SD	*n*/N	Mean Value ± SD	*n*/N	Mean Value ± SD	*n*/N
Time of assessment (months)	5.9 (SD ± 4.9)	31/40	9.5 (SD ± 5.1)	29/40	93 (SD ± 71.7)	31/40
Average daily stool frequency	1.4 (SD ± 1.9)	28/40	1.4 (SD ± 1.7)	28/40	2.0 (SD ± 3.6)	38/40
Leukocytes (×10^3^/µL)	5.7 (±2.5)	30/40	5.8 (±2.3)	28/40	6.5 (±3.6)	40/40
CRP (mg/dL)	2.6 (±4.6)	28/40	1.6 (±2.1)	28/40	3.8 (±5.7)	36/40
**Categorical Variables**	***n*** ** (%) or Mean ± SD**	***n*/N**	***n* (%) or Mean ± SD**	***n*/N**	***n* (%) or Mean ± SD**	***n*/N**
Abdominal Pain Score		29/40		28/40		39/40
0 = No pain	20 (69.0)		26 (92.9)		29 (74.4)	
1 = Mild pain	8 (27.6)		1 (3.6)		3 (7.7)	
2 = Moderate pain	1 (3.4)		0 (0)		7 (17.9)	
3 = Severe pain	0 (0.0)		1 (3.6)		0 (0)	
Clinical remission (PRO2) (SF ≤ 3 and APS ≤ 1)		29/40		28/40		39/40
Yes	26 (89.7)		24 (85.7)		32 (82.1)	
No	3 (10.3)		4 (14.3)		7 (17.9)	
Endoscopy		20/40		28/40		31/40
0 = Normal	11 (55)		9 (60)		14 (45.2)	
1 = Mild	4 (20)		5 (33.3)		10 (32.3)	
2 = Moderate	4 (20)		0 (0)		5 (16.1)	
3 = Severe	1 (5)		1 (6.7)		2 (6.5)	

**Table 3:** Inflammatory bowel disease (IBD)-specific medical therapy in Crohn’s disease patients before, 3–18 months after, and during long-term follow-up after liver transplantation (LT).

	Before LT (3–6 m Pre-LT)		After LT (3–18 m Post-LT)		During Long-Term Follow-Up (>18 m Post-LT)	
	*n* (%)	*n*/N	*n* (%)	*n*/N	*n* (%)	*n*/N
General anti-inflammatory drugs						
Aminosalicylates	11 (35.3)	30/40	6 (21.4)	28/40	19 (47.5)	40/40
Steroids	12 (40)	30/40	20 (71.4)	28/40	23 (57.5)	40/40
Immunosuppressive Therapy for LT				28/40		40/40
Tacrolimus			9 (32.1)		18 (45)	
Ciclosporin			1 (3.6)		4 (10)	
MMF			1 (3.6)			
Combinations			17 (60.7)		18 (45)	
Advanced CD therapies	4 (11.7)	34/40	1 (3.6)	28/40	3 (7.5)	40/40
Anti-TNF	3 (8.8)		1 (3.6)		2 (5.0)	
Vedolizumab					1 (2.5)	
Combination therapies	1 (2.9)					

**Table 4:** Endoscopic disease activity of Crohn’s disease patients at long-term follow-up (>18 months after liver transplantation).

Endoscopy	(*n* = 31)	%
0 = Mucosal healing	14	45.2
1 = Mild disease activity	10	32.3
2 = Moderate disease activity	5	16.1
3 = Severe disease activity	2	6.5

## Data Availability

The data presented in this study are available upon request from the corresponding author. The data are not publicly available due to the confidentiality of patient data.

## Supplementary Materials

The following supporting information can be downloaded at: https://www.mdpi.com/article/10.3390/biomedicines13092200/s1, Table S1: Clinical remission rates were assessed at two post-transplant time intervals (3–18 months and >18 months), comparing patients aged <40 years with those aged ≥40 years. No statistically significant differences in remission rates were observed between the two age groups at either time point.

## Abbreviations

The following abbreviations are used in this manuscript:

APS	Abdominal pain score
CD	Crohn’s disease
CRP	C-reactive protein
fCal	Fecal calprotectin
IBD	Inflammatory bowel disease
LT	Liver transplantation
PSC	Primary sclerosing cholangitis
SF	Stool frequency
UC	Ulcerative Colitis
