# Supplementary material for: Long-Term Clinical and Endoscopic Outcomes of Crohn’s Disease Following Liver Transplantation: A Multicenter Cohort Study
Source: Biomedicines. 2025 Sep 8;13(9):2200. doi: 10.3390/biomedicines13092200 (PMC12467632; doi:10.3390/biomedicines13092200)
Supplement: Supplementary file 1 [file biomedicines-13-02200-s001.zip › biomedicines-3803970-supplementary.pdf]

Supplementary Table S1.

	**Clinical Remission**		**No clinical Remission**		
age (y)	< 40	> 40	< 40	> 40	*p*-value
3-18 months after LT (26/30)	9	14	1	2	0.846
Long-term follow-up (28/30)	7	15	1	5	0.466

Clinical remission rates were assessed at two post-transplant time intervals (3–18 months and >18 months), comparing patients aged <40 years with those aged ≥40 years. No statistically significant differences in remission rates were observed between the two age groups at either time point.
